# Nanostructured Lipoxin A4: Understanding Its Biological Behavior and Impact on Alzheimer’s Disease (Proof of Concept)

**DOI:** 10.3390/pharmaceutics17050649

**Published:** 2025-05-15

**Authors:** Natália Cristina Gomes-da-Silva, Isabelle Xavier-de-Britto, Marilia Amável Gomes Soares, Natalia Mayumi Andrade Yoshihara, Derya Ilem Özdemir, Eduardo Ricci-Junior, Pierre Basílio Almeida Fechine, Luciana Magalhães Rebelo Alencar, Maria das Graças Muller de Oliveira Henriques, Thereza Christina Barja-Fidalgo, Cristian Follmer, Ralph Santos-Oliveira

**Affiliations:** 1 Laboratory of Nanoradiopharmacy and Synthesis of New Radiopharmaceuticals, Nuclear Engineering Institute, Brazilian Nuclear Energy Commission, Rio de Janeiro 21941906, RJ, Brazil; nataliacristinasilva@id.uff.br (N.C.G.-d.-S.); isabellexbritto@gmail.com (I.X.-d.-B.); marilia.agsoares@gmail.com (M.A.G.S.); natalia.yoshihara@gmail.com (N.M.A.Y.); 2 Faculty of Pharmacy, Department of Radiopharmacy, Ege University, Bornova, Izmir 35040, Turkey; derya.ilem@ege.edu.tr; 3 School of Pharmacy, Federal University of Rio de Janeiro, Rio de Janeiro 21941900, RJ, Brazil; ricci@pharma.ufrj.br; 4 Group of Chemistry of Advanced Materials (GQMat), Department of Analytical Chemistry and Physical-Chemistry, Federal University of Ceará, Fortaleza 451970, CE, Brazil; fechine@ufc.br; 5 Biophysics and Nanosystems Laboratory, Department of Physics, Federal University of Maranhão, São Luis 65065690, MA, Brazil; luciana.alencar@ufma.br; 6 Laboratory of Cellular & Molecular Pharmacology, Department of Cell Biology, Instituto de Biologia Roberto Alcantara Gomes (IBRAG), Universidade do Estado do Rio de Janeiro, Rio de Janeiro 20551030, RJ, Brazil; gracamohenriques@gmail.com (M.d.G.M.d.O.H.); cbarja.uerj@gmail.com (T.C.B.-F.); 7 Laboratory of Biological Chemistry of Neurodegenerative Disorders, Department of Physical Chemistry, Institute of Chemistry, Federal University of Rio de Janeiro, Rio de Janeiro 21941909, RJ, Brazil; crisfollmer@gmail.com; 8 Laboratory of Radiopharmacy and Nanoradiopharmaceuticals, Rio de Janeiro State University, Rio de Janeiro 23070200, RJ, Brazil

**Keywords:** neurodegenerative, nanotechnology, nanoparticles, neurology

## Abstract

**Background/Objectives**: Lipoxins, particularly Lipoxin A4 (LXA4), are endogenous lipid mediators with potent anti-inflammatory and pro-resolving properties, making them promising candidates for the treatment of inflammatory and neurodegenerative disorders. However, their therapeutic application is limited by poor stability and bioavailability. This study aimed to develop and characterize nanomicelles encapsulating LXA4 (nano-lipoxin A4) to improve its pharmacological efficacy against Alzheimer’s disease (AD), a neurodegenerative condition marked by chronic inflammation and beta-amyloid (Aβ) accumulation. **Methods**: Nano-lipoxin A4 was synthesized using Pluronic F-127 as a carrier and characterized in terms of morphology, physicochemical stability, and in vitro activity against Aβ fibrils. Dissociation of Aβ fibrils was assessed via Thioflavin-T fluorescence assays and transmission electron microscopy. In vivo biodistribution and pharmacokinetic profiles were evaluated using technetium-99m-labeled nano-lipoxin A4 in rodent models. Hepatic biochemical parameters were also measured to assess potential systemic effects. **Results**: In vitro studies demonstrated that nano-lipoxin A4 effectively dissociated Aβ fibrils at concentrations of 50 nM and 112 nM. Electron microscopy confirmed the disruption of fibrillar structures. In vivo imaging revealed predominant accumulation in the liver and spleen, consistent with reticuloendothelial system uptake. Pharmacokinetic analysis showed a prolonged half-life (63.95 h) and low clearance rate (0.001509 L/h), indicating sustained systemic presence. Biochemical assays revealed elevated liver enzyme levels, suggestive of increased hepatic metabolism or potential hepatotoxicity. **Conclusions**: Nano-lipoxin A4 exhibits significant therapeutic potential for Alzheimer’s disease through effective modulation of Aβ pathology and favorable pharmacokinetic characteristics. However, the elevation in liver enzymes necessitates further investigation into systemic safety to support clinical translation.

## 1. Introduction

Lipoxins are a family of bioactive lipid mediators derived from arachidonic acid, an essential fatty acid that is a component of cell membrane phospholipids [1]. First discovered in the 1980s, lipoxins play a crucial role in the resolution phase of inflammation, acting as endogenous anti-inflammatory and pro-resolving agents [2,3]. Unlike other eicosanoids, such as prostaglandins and leukotrienes, which typically promote inflammation, lipoxins help to terminate inflammatory responses and restore tissue homeostasis [4].

The lipoxins are produced through the action of lipoxygenase enzymes, specifically 5-lipoxygenase and 15-lipoxygenase. These enzymes catalyze the oxygenation of arachidonic acid to generate intermediate metabolites that are further processed into lipoxins. The two primary lipoxins, Lipoxin A4 (LXA4) and Lipoxin B4 (LXB4), are characterized by a unique triene structure, which includes a conjugated double bond system. This distinct chemical structure is critical for their biological activity and receptor binding [5,6,7,8].

In terms of biological functions and mechanisms of action, the lipoxins exert their effects by binding to specific receptors, such as the formyl peptide receptor 2 (FPR2/ALX), which are expressed on various immune cells, including neutrophils, macrophages, and epithelial cells. Upon receptor engagement, lipoxins modulate immune cell function in several ways: (i) Inhibition of Neutrophil Chemotaxis and Adhesion: Lipoxins reduce the recruitment of neutrophils to the site of inflammation and inhibit their adhesion to the endothelium, thereby limiting tissue damage; (ii) Promotion of Macrophage Phagocytosis: They enhance the clearance of apoptotic cells and cellular debris by macrophages, a process known as efferocytosis, which is essential for the resolution of inflammation; and (iii) Reduction in Pro-inflammatory Cytokines: Lipoxins suppress the production of pro-inflammatory cytokines and chemokines, thereby attenuating the inflammatory response [9,10,11,12].

Given their potent anti-inflammatory and pro-resolving properties, lipoxins have garnered significant interest as potential therapeutic agents for treating chronic inflammatory diseases, such as asthma, cardiovascular diseases, COVID-19, and autoimmune disorders [10,13,14,15,16,17]. Synthetic analogs of lipoxins and strategies to enhance endogenous lipoxin production are under investigation as potential treatments to modulate excessive inflammation and promote tissue healing.

Lipoxins, despite their potent anti-inflammatory and pro-resolving actions, face significant challenges regarding their stability and bioavailability in vivo. This instability is a critical factor that limits their therapeutic potential and necessitates further research into stabilization strategies. The main factors that contribute to lipoxin instability are as follows: (i) Rapid Metabolism: Lipoxins are rapidly metabolized in the body, primarily by enzymes such as 15-hydroxyprostaglandin dehydrogenase and 12-hydroxydehydrogenase. These enzymes convert lipoxins into inactive metabolites, significantly reducing their bioactive lifespan. This rapid degradation is a major challenge to harnessing their therapeutic benefits. (ii) Short Half-Life: The in vivo half-life of lipoxins is relatively short, often measured in minutes. This short half-life limits their ability to exert sustained biological effects, which is crucial for the resolution of inflammation in chronic conditions. (iii) Chemical Instability: The chemical structure of lipoxins, which includes conjugated double bonds, makes them susceptible to oxidation and other forms of chemical degradation. This instability can lead to the loss of biological activity and the formation of inactive or potentially harmful degradation products [18,19,20,21].

To overcome these challenges and enhance the therapeutic potential of lipoxins, several strategies are being explored. In this direction, the development of synthetic analogs of lipoxins, known as lipoxin analogs, which are designed to resist enzymatic degradation and maintain bioactivity for longer periods, are being investigated [22]. These analogs often involve modifications to the native lipoxin structure to improve stability while preserving receptor binding and function. Another strategy is encapsulation techniques, such as using liposomes or nanoparticles, which are being investigated to protect lipoxins from rapid metabolism and degradation [23]. These delivery systems can also enhance the targeted delivery of lipoxins to specific tissues or sites of inflammation, improving their therapeutic efficacy. Another approach involves strategies to enhance the endogenous production of lipoxins in the body. This can be achieved through dietary interventions, pharmacological agents, or genetic modifications that increase the activity of lipoxygenase enzymes involved in lipoxin biosynthesis.

In the context of Alzheimer’s disease (AD), a neurodegenerative disorder characterized by progressive cognitive decline, the role of neuroinflammation has become increasingly recognized as a central pathological mechanism. Chronic neuroinflammation, driven by microglial and astrocytic activation, exacerbates the accumulation of beta-amyloid (Aβ) plaques and hyperphosphorylated tau, two hallmark features of AD pathology. While acute inflammation can initially serve protective functions, its failure to resolve properly in AD perpetuates neuronal injury and synaptic dysfunction [24,25,26,27].

Recent studies have highlighted the therapeutic potential of Lipoxin A4 in modulating neuroinflammation and mitigating disease progression in Alzheimer’s pathology. By binding to the ALX/FPR2 receptor (Lipoxin A4 receptor), LXA4 exerts its pro-resolving effects, including (i) the inhibition of pro-inflammatory cytokine release (e.g., TNF-α, IL-1β, IL-6), (ii) reductions in microglial and astrocytic hyperactivation, (iii) the promotion of phagocytosis of beta-amyloid plaques, and (iv) the protection of neuronal integrity by preventing oxidative stress and apoptosis. Furthermore, emerging data suggest that LXA4 can regulate the balance between neuroinflammation and its resolution by enhancing the clearance of toxic protein aggregates, thereby attenuating the progression of cognitive decline [13,28].

In this sense, this study has developed, fully characterized, and evaluated the application of nano-lipoxin A4 in vitro and in vivo.

## 2. Materials and Methods

### 2.1. Reagents and Solvents

All reagents and solvents used in the experiments, including Lipoxin A4 (C_20_H_32_O_5_), were purchased from Sigma-Aldrich (St. Louis, MO, USA).

### 2.2. Formulation Lipoxin

Pluronic F-127 (PF-127) micellar dispersions were prepared by the cold method. Specifically, PF-127 was dissolved in ultrapure water at a concentration of 20% *w/v* and allowed to hydrate overnight at 4 °C under gentle stirring to ensure complete dissolution. This solution forms micelles spontaneously upon reaching the critical micellization temperature, typically around 20–25 °C. Lipoxin A4 (LXA4), dissolved in ethanol, was added to the cold PF-127 dispersion to achieve a final concentration of 6 μg per 1.2 mL. The resulting mixture was subjected to ultrasonic homogenization using a UP100H ultrasonic processor (Hielscher Ultrasonics, Teltow, Germany) at 100% amplitude and 1 cycle for 5 min while maintaining a bath temperature of 1 °C using a thermocycler (SolidSteel^®^, model SSdu). The final formulation was stored at 2–8 °C to preserve its physicochemical stability.

## 3. Characterization

### 3.1. Scanning Electron Microscopy (SEM)

Nano-lipoxin A4 was examined using a TESCAN Clara FEG BrightBeam electron column microscope (TESCAN ORSAY Holding SA, Brno, Czech Republic) operating at a voltage of 5 kV from Oxford Instruments. Sample preparation involved diluting the micelles in ultrapure water, spotting them on the stub surface with carbon tape, and characterizing them using Oxford Instruments AZTec 6.1 EDS software. Scanning electron microscopy is the method of choice for microstructural characterization due to its versatility, allowing analysis at multiple scales from millimeters to nanometers and providing micrographs, thus offering information on the surface morphology and size of the nanoparticles.

### 3.2. Fibrilation In Vitro Experimentation 

#### Fibrillation and Fibril-Dissolving Assay

To analyze the fibril-destabilizing activity, 50 μM of preformed fibrils of Aβ1-42 (10 mM sodium phosphate, pH 7.4, 100 mM NaCl plus 5 μM ThT) was incubated in the presence of 50 and 112 nM of Lipoxin nanomicelles or an equivalent concentration of Pluronic F127 (nanomicelle control), and the ThT fluorescence was monitored over the time course at 25 °C, without agitation. In the case of Lipoxin (dissolved in 100% ethanol), an equivalent concentration of DMSO was used as the control. To evaluate the inhibitory activity of Lipoxin nanomicelles or Lipoxin on the fibrillation of Aβ1-42, 50 μM of the protein monomer, in the presence of 5 μM of ThT, was incubated in the presence of 280 nM of Lipoxin or ethanol at 37 °C and 450 rpm in a 96-well microplate using thermomixer equipment (Eppendorf, Hamburg, Germany).

### 3.3. In Vivo Experimentation

#### 3.3.1. Animal

All animal experiments were conducted following approval by the Animal Care and Use Committee of the Universidade do Estado do Rio de Janeiro (protocol CEUA/8059100220/2021), in accordance with the Guide for the Care and Use of Laboratory Animals (National Research Council, 1996, Washington, D.C., United States).

A total of 12 male Balb/c mice (4 to 6 weeks old, weighing 20–30 g) were used. Animals were housed individually in ventilated cages under controlled environmental conditions (temperature: 21.0 ± 1.0 °C; 12:12 h light–dark cycle) with unrestricted access (ad libitum) to food and water.

The animals were divided into two major experimental blocks. For radiopharmacokinetic and biodistribution analysis, six mice were allocated into two subgroups (*n* = 3 each), with sample collection at 2 h and 24 h post-administration of the 99mTc-labeled nano-lipoxin A4. The remaining six animals were used for biochemical evaluations, comprising the treated (*n* = 3) and control (*n* = 3) groups. Blood specimens for biochemical analysis were obtained via cardiac puncture 24 h post-intraperitoneal administration of the nanoformulation.

#### 3.3.2. Animal Preparation

Animals were anesthetized by intraperitoneal injection (ketamine 100 mg·kg^−1^ and xylazine 20 mg·kg^−1^).

### 3.4. Radiolabeling of Lipoxin A4 Nanomicelles

The process of labeling with technetium-99 was performed directly by adding 1 mL of stannous chloride (SnCl_2_, 80 µL/mL) (Sigma-Aldrich), combined with 1 mL of technetium-99m (^99m^Tc) with an activity of 544 μCi, followed by a 10 min incubation period. Subsequently, 15 µg o nano-lipoxin A4 nanomicelles was incorporated into this mixture and subjected to a further 10 min incubation to label the structures.

### 3.5. Radio Thin Layer Chromatography

To confirm the efficacy of the radiolabeling process, Radio Thin Layer Chromatography (RTLC) was performed using Whatman paper No. 1 using 2 μL of 99mTc micelles and acetone (Sigma-Aldrich) as the mobile phase. The radioactivity of the strips was checked in a γ counter (Hidex, Turku, Finland). RTLC was performed in triplicate for each time point.

### 3.6. Biodistribution/Tissue Deposition Assay

For the biodistribution/tissue deposition studies, 27.2 μCi (**1.0** MBq)/0.2 mL of ^99m^Tc-nano-lipoxin A4 was injected intraperitonially (i.p.), evaluating the systemic behavior in healthy animals. Animals were sacrificed 24 h post-injection by using an excess of anesthesia (isoflurane chamber); the blood and organs of interest (heart, brain, stomach, intestine, bladder, kidney (right and left), lung (right and left), liver, and spleen) were immediately dissected out and weighed for quantitative estimation of gamma counts using a gamma counter (Hidex, Turku, Finland). The results were expressed as percentage of injected dose per organ (%ID/g).

### 3.7. Pharmacokinetic Analysis

For pharmacokinetic analysis, mice (*n* = 6) received a dose of ^99m^Tc-nano-lipoxin A4 with an activity of 31.6 uCi/1169.2 Mbq per animal, injected intraperitonially (i.p.), Subsequently, 2 µL of caudal blood was collected every hour at the following time points: 0 h, 1 h, 2 h, 3 h, 5 h, 24 h. Blood samples were read on an automatic gamma particle counter (Hidex) to quantify the percentage of activity injected per gram of tissue (%AI/g).

For radiopharmacokinetics studies, the Balb/c mice received 1 µg of radiolabeled Lipoxin A4 nanomicelles (^99m^Tc-nano-lipoxin A4) administered by intraperitoneal injection. Subsequently, 2 µL of blood samples were collected from the tail vein at the following time points: 0, 1, 2, 3, 5, and 24 h. The calculated pharmacokinetic parameters were (i) zero-time concentration (C_0_), (ii) elimination constant (K_e_), (iii) volume distribution (V_d_), (iv) clearance (CL), and (v) half-life elimination (t_1/2_). The radioactive count conversion to Lipoxin A4-loaded nanomicelle mass was calculated considering the initial mass of 1 µg using Equation (1).

(1) $$N=N_{o}e^{-ƛt}$$

It is important to note that the pharmacokinetic profile presented exclusively reflects the behavior of the radiolabeled formulation and the endogenous Lipoxin A4 was not considered.

### 3.8. Biochemical Analysis

Blood specimens were obtained through cardiac puncture from healthy mice that received an intraperitoneal injection of nano-lipoxin A4, comprising the intervention group, 24 h after administration (with a sample size of *n* = 6 per group). Subsequently, 0.5 mL of these blood samples was transferred into microtubes pre-filled with 0.5 mL of the anticoagulant Heparin (sourced from Sigma-Aldrich, Sao Paulo, Brazil). To separate the plasma, the samples underwent centrifugation at 5000 rpm for 5 min at a temperature of 4 °C (IKA G-L SO32, Stafen, Germany). Following separation, the plasma samples were processed in alignment with the protocols provided by the manufacturer (MaxBio Auto 100, GENRUI Biotech INC, Shenzhen, China). This processing aimed to assess the enzymatic activities of several key biomarkers: alanine aminotransferase (ALT), aspartate aminotransferase (AST), gamma GT (GGT), lactate dehydrogenase pyruvate (LDH-P), cholesterol (CHOL), lipase D (LPS), glucose (GLU), and alpha amylase (AMS).

### 3.9. Statistical Analyses

Both the in vitro and in vivo experiments were performed in triplicate or quadruplicate; values are expressed as means ± SD. Differences between groups were tested for significance by one-way ANOVA followed by Turkey’s multiple comparisons test, using GraphPad Prism 8.1 software. A *p*-value ≤ 0.05 was considered significant.

## 4. Results

### 4.1. Scanning Electron Microscopy

**Figure 1:**
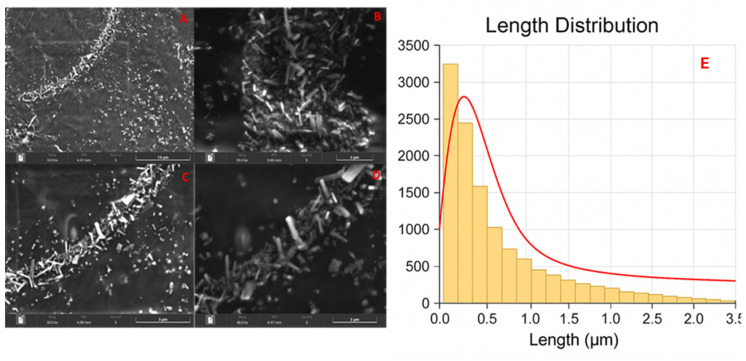
SEM images. The image consists of four scanning electron microscope (SEM) micrographs labeled (**A**–**D**), showing increasing magnifications from 10,000× (**A**) to 40,000× (**D**), with scale bars indicating 10 µm, 2 µm, 5 µm, and 2 µm, respectively. The micrographs reveal a high density of eneedle-like crystalline structures aligned along the boundary. Panels (**A**–**D**): scanning electron microscopy (SEM) images of the synthesized nanoformulation at various magnifications. Rod-shaped particles are evident with consistent anisotropic morphology across fields. Scale bars are indicated on each micrograph. Panel (**E**): length distribution histogram of the nanoparticles (*n* ≈ 3700), extracted via ImageJ software v8.0 from SEM micrographs. The superimposed curve represents a log-normal fit to the data. The size distribution is characterized by a geometric mean length of 112 nm and a geometric standard deviation of 1.75, indicating moderate polydispersity and confirming the non-Gaussian nature of the population.

The morphology of nano-lipoxin A4 was characterized by their length, diameter, and aspect ratio (length/diameter), revealing significant variability in particle dimensions. Figure 1 shows the length of lipoxin A4 nanomicelles exhibited a mean value of 65 nm, with a standard deviation of 117 nm. The diameter showed a mean value of 41 nm, with a standard deviation of 58 nm. These results suggest a predominant presence of shorter and narrower particles, with a minority of highly elongated structures as outliers. The length histogram highlighted a concentration of particles within the smaller length ranges, suggesting that most particles adopt compact or moderately elongated morphologies. Likewise, the diameter distribution emphasized the prevalence of narrow particles, with some larger ones reflecting the variability of the synthesis.

Aspect ratio, defined as the ratio between length and diameter, is a key metric for assessing particle uniformity and morphology. The mean aspect ratio of 1.54, coupled with a standard deviation of 1.38, indicates moderate uniformity. The majority of particles exhibit low aspect ratios, corresponding to compact or slightly elongated structures. However, outliers with higher aspect ratios signify the presence of rod-shaped or highly elongated particles. The aspect ratio histogram confirmed a dominance of low values, reflecting the compact nature of most particles, while a smaller fraction of particles exhibited elongated geometries.

### 4.2. Fibrilation In Vitro Experimentation Fibrillation Assay for Aβ Analysis

**Figure 2:**
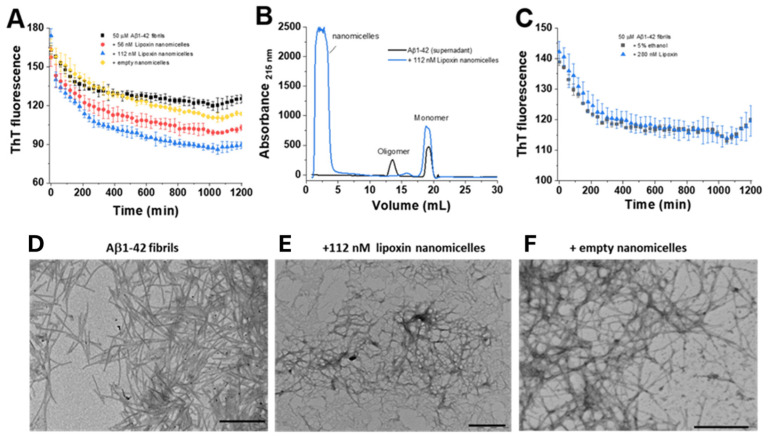
In vitro test. Dissociative analysis of beta-amyloid peptides by treatment with nano-lipoxin A4. (**A**) ThT kinetics with a maximum time of 1200 min with samples treated with 50 nM and 112 nM of filled nanomicelles. (**B**) Spectrophotometric analysis of the AB supernatant in the presence or absence of nano-lipoxin A4. (**C**) ThT fluorescence of peptides treated with pure lipoxin. (**D**–**F**) SEM images for morphological analysis of the structures.

To evaluate the effects of nano-lipoxin A4 on the dissolution process of beta-amyloid peptides, Thioflavin-T(ThT) kinetics were carried out using a time of 1200 min (20 h) in the presence and absence of nanomicelles (Figure 2A). Our results show that treatment with 50 and 112 nM nano-lipoxin A4 had a significant effect on peptide reduction when compared to empty nanomicelles and the untreated control (Aβ 1-42).

In addition, we evaluated the formation of oligomers and monomers from the supernatant of Aβ samples upon treatment with lipoxin nanomicelles. Figure 2B shows an increase in the formation of monomers when exposed to nano-lipoxin A4 in the higher concentration (112 nM), indicating a dissociative effect of the oligomers and monomers formation when compared to the Aβ samples alone.

As a control criterion, we evaluated the ability of lipoxin alone to modulate the behavior of Aβ peptides via ThT kinetics. Figure 2C shows that lipoxin alone is unable to reduce Aβ dissociation, suggesting that the nanostructured treatment has a real effect on fiber elimination.

To check the morphology of the AB structures after treatment with lipoxin nanomicelles, we performed scanning electron microscopy (SEM). We noticed a visual change in the morphology of the structures treated with nano-lipoxin A4 when compared to the empty nanomicelles.

### 4.3. In Vivo Biodistribution: Tissue Deposition

**Figure 3:**
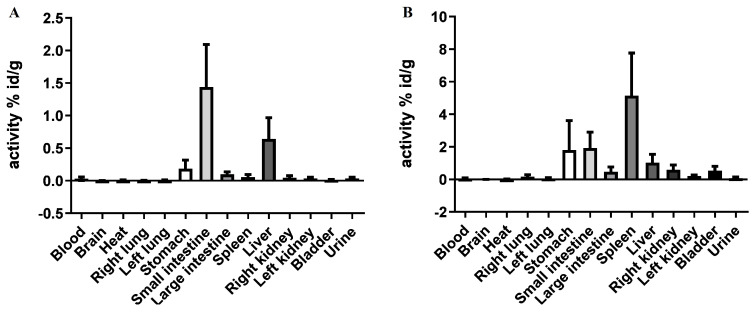
The biodistribution (tissue deposition assay) shows the present behavior of nano-lipoxin A4 in all organs. (**A**) represents the 2 h biodistribution and (**B**) the 24 h biodistribution.

The biodistribution/tissue deposition assay 2 and 24 h post-injection is expressed in Figure 3.

The biodistribution/tissue deposition assay showed a high spleen uptake at both 2 and 24 h post-injection. Also, the liver showed a moderate uptake in both analyses, indicating that nano-lipoxin A4 accumulates in the reticuloendothelial system (RES), consistent with the behavior of nanoparticles. A minimal accumulation in blood suggests that the nanomicelles are rapidly cleared from circulation and preferentially taken up by specific organs.

### 4.4. Radiolabeling Quality Control

The results of the radiolabeling quality control are expressed in Table 1:

The radiolabeling efficacy reaches its peak at 2 h (99.73% ± 0.07), demonstrating optimal labeling conditions. Over 24 h, there is a slight reduction (~7.4% decrease) in radiolabeling efficacy, which may indicate a minor instability or breakdown of the radiolabel. It is important to note that the standard deviations are consistently low after 1 h, highlighting the high reproducibility of the results.

### 4.5. Radiopharmacokinetics

**Figure 4:**
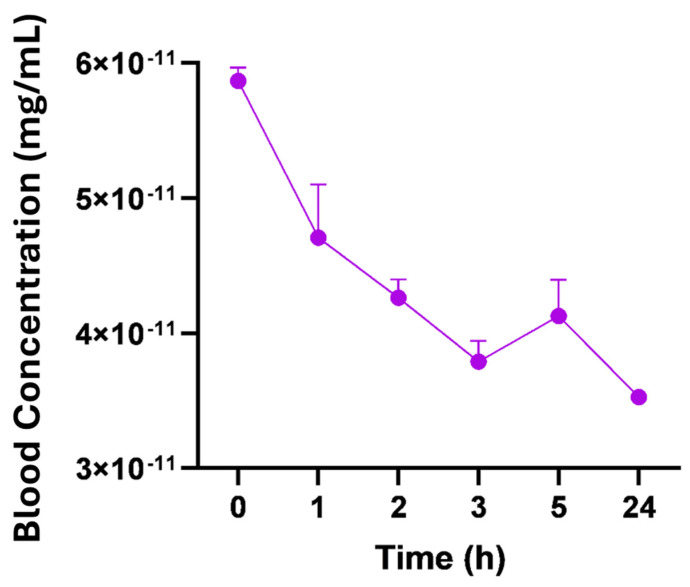
Plasma concentration–time curve following intraperitoneal administration of nano-lipoxin A4 labeled with [99mTc] in healthy mice from 0 to 24 h. The graphs show the mean ± SEM (*n* = 3).

To evaluate the pharmacokinetic profile and bloodstream permanence of [99mTc]-labeled nano-lipoxin A4, we conducted pharmacokinetic tests, as shown in Figure 4. Blood samples (2 μL) were collected from the tail vein at various time points following intraperitoneal injection. The results showed a high concentration of nanomicelles immediately post-injection (time 0 h), followed by a gradual decline over the first 1–3 h.

The quantitative analysis of the PK of nano-lipoxin A4 is resumed in Table 2.

It is observed that the initial concentration was 4.700 × 10^−11^ mg/mL, with an elimination rate constant of 0.0119 h^−1^. The volume of distribution was 0.1282 L, and the elimination half-life was 2.664 days, with a clearance rate of 0.001509 L/h. The high elimination half-life (63.95 h or ~2.66 days) combined with a low clearance rate (0.001509 L/h) indicates slow elimination of nano-lipoxin A4, suggesting a prolonged presence in the system, which may be beneficial for sustained therapeutic effects. Also, the low volume of distribution (128.2 mL or 0.1282 L) suggests that nano-lipoxin A4 remains largely confined to the central compartment (blood/plasma) and does not distribute extensively into tissues.

### 4.6. Biochemical Analysis

The biochemical results are expressed in Table 3, which presents the results of healthy mice treated with 0.2 mL of nano-lipoxin A4 administered via intraperitoneal injection. The treatment with nano-lipoxin A4 led to significant changes in various biochemical markers, particularly those related to liver function (ALT, AST, GGT), tissue injury (LDH-P), lipid metabolism (CHOL, LPS), and glucose regulation (GLU). The elevated levels of these markers indicate that the treatment may induce stress or damage in multiple organs, warranting further investigation into the safety and systemic effects of Lipoxin A4 nanomicelles.

## 5. Discussion

In this study, we developed and characterized Lipoxin A4-loaded nanomicelles (nano-lipoxin A4) and demonstrated their ability to modulate amyloid-beta (Aβ) fibrillation, a critical hallmark of Alzheimer’s disease (AD).

The morphological characteristics of nano-lipoxin A4 significantly influence their functional properties in biomedical applications. Variability in length, diameter, and aspect ratio affects critical parameters such as surface area-to-volume ratio, stability, biological interactions, and therapeutic efficacy. Smaller nanoparticles with higher aspect ratios exhibit an increased surface area-to-volume ratio, enhancing their interaction with biological membranes. This property facilitates more efficient drug encapsulation and release dynamics, which are essential for the therapeutic potential of nano-lipoxins [29].

However, the presence of elongated particles may increase the likelihood of aggregation due to interparticle forces such as van der Waals attractions. Aggregation impacts their stability, reactivity, and behavior in biological systems, including transport across membranes and uptake into cells. Stabilizing agents or surface modifications may therefore be required to maintain colloidal stability and prevent particle agglomeration during storage and administration [30,31,32].

Regarding biological interactions, compact particles with smaller aspect ratios tend to diffuse more evenly in biological environments, which is advantageous for systemic distribution. In contrast, elongated rod-shaped structures may provide unique benefits, such as enhanced membrane penetration and targeted delivery, due to their anisotropic morphology. These shape-dependent interactions are critical for optimizing nanoparticle design for specific therapeutic targets [33,34].

Finally, optimizing nano-lipoxin particle dimensions is essential to achieve a balance between dispersibility, stability, and bioavailability. Tailoring particle sizes and shapes to specific therapeutic needs can maximize their efficacy, particularly in drug delivery systems where these parameters directly influence pharmacokinetics and biodistribution [35,36,37,38].

Furthermore, this study provides insights into the biological behavior, pharmacokinetics, biodistribution, and systemic effects of nano-lipoxin A4, reinforcing its therapeutic potential while addressing challenges related to its stability and bioavailability.

The in vitro findings revealed a significant dissociative effect of nano-lipoxin A4 on Aβ fibrils, as evidenced by a reduction in Thioflavin-T (ThT) fluorescence, which correlates with fibril formation. Treatment with nano-lipoxin A4 at concentrations of 50 nM and 112 nM effectively destabilized Aβ fibrils, promoting their dissociation into oligomers and monomers. These results were further corroborated by spectrophotometric analysis and electron microscopy, which showed clear morphological changes in Aβ aggregates. Importantly, free Lipoxin A4 (LXA4) alone did not achieve this effect, underscoring the role of the nanomicellar formulation in enhancing LXA4 stability and bioavailability. Similar studies have demonstrated that nanoparticle-based delivery systems improve the therapeutic activity of bioactive molecules by enhancing their stability, targeting capabilities, and biodistribution profile, particularly for molecules like lipoxins that are susceptible to rapid metabolism and degradation in vivo [39,40].

These findings are particularly significant given the central role of Aβ aggregation in AD pathogenesis. Aβ fibrils and oligomers are known to drive neurotoxicity, synaptic dysfunction, and neuroinflammation, which perpetuate disease progression [41]. Strategies that can destabilize fibrils and reduce Aβ load have been shown to alleviate neurodegeneration and improve cognitive outcomes in AD models [42]. The dissociative effects of nano-lipoxin A4 observed here suggest its potential to mitigate Aβ-related pathology and its downstream consequences, including chronic neuroinflammation.

The biodistribution analysis revealed a preferential accumulation of nano-lipoxin A4 in the spleen and liver at both 2 and 24 h post-injection, consistent with the uptake of nanoparticles by the reticuloendothelial system (RES) [43]. The spleen plays a crucial role in immunomodulation, and its high uptake of nano-lipoxin A4 could support its role in reducing systemic and neuroinflammatory responses. Additionally, the moderate liver uptake suggests hepatic clearance and metabolism, which aligns with the lipid-soluble nature of LXA4 and the nanomicellar formulation [44]. The minimal blood accumulation observed indicates rapid clearance from circulation, reflecting the efficiency of the nanocarrier system. This biodistribution pattern mirrors findings from other nanocarrier studies, where nanoparticle accumulation in the RES and liver is commonly observed due to macrophage uptake [45].

The pharmacokinetic results demonstrated a prolonged elimination half-life of approximately 63.95 h (2.66 days) and a low clearance rate of 0.001509 L/h. These parameters suggest that nano-lipoxin A4 exhibits slow elimination and prolonged systemic presence, which is advantageous for sustained therapeutic effects. Lipoxin A4 in its native form is known to have a short half-life due to rapid enzymatic degradation [46]. Nanomicellar encapsulation significantly overcomes this limitation, providing stability and extending its pharmacological activity. This prolonged circulation time could enable nano-lipoxin A4 to exert sustained effects on neuroinflammation and Aβ clearance, both of which are critical targets in AD management [47].

The biochemical analysis showed elevated levels of liver enzymes, such as alanine aminotransferase (ALT), aspartate aminotransferase (AST), and gamma-glutamyl transferase (GGT), along with increased lactate dehydrogenase (LDH), cholesterol, lipase, and glucose. These findings suggest an increased metabolic burden on the liver, likely due to the uptake and clearance of nano-lipoxin A4. Elevated liver enzymes are often indicative of hepatic stress or injury, which has been observed in other nanoparticle-based formulations due to their metabolism and interaction with liver cells [48]. The increased glucose and lipid markers could reflect heightened energy demands associated with the processing of nano-lipoxin A4, particularly within hepatic and metabolic pathways [49]. While these effects are not uncommon for lipid-based delivery systems, further studies are required to optimize the formulation to minimize hepatic stress while preserving therapeutic efficacy.

The implications of these findings are substantial in the context of Alzheimer’s disease. Neuroinflammation, driven by microglial and astrocytic activation, plays a central role in AD pathogenesis, exacerbating Aβ accumulation and neuronal dysfunction [50]. Lipoxin A4 is a well-recognized pro-resolving lipid mediator that modulates inflammation by inhibiting the release of pro-inflammatory cytokines (e.g., TNF-α, IL-1β, IL-6) and promoting the clearance of apoptotic cells and toxic aggregates [51]. Decreased LXA4 levels in cerebrospinal fluid have been correlated with cognitive decline in AD patients [51]. Our findings suggest that the nanostructured formulation of LXA4 not only enhances its stability and bioavailability but also allows it to effectively interfere with Aβ fibrillation, a key driver of AD pathology.

It is important to note that free Lipoxin A4 is notoriously expensive due to the intricate and resource-intensive synthesis process required to produce its biologically active stereoisomer with high purity. In Brazil, the costs associated with acquiring sufficient quantities of free Lipoxin A4 are disproportionately high, limiting its use in large-scale or comparative studies, and for that reason we have not intercompared the free Lipoxin A4 with the nanostructured assuming a proof-of-concept study.

In conclusion, this study highlights the potential of nanostructured Lipoxin A4 as a promising therapeutic strategy for Alzheimer’s disease by addressing two key pathological features: Aβ fibrillation and neuroinflammation. The in vitro dissociative effects on Aβ fibrils, combined with favorable pharmacokinetics and targeted biodistribution, support its therapeutic utility. However, the observed biochemical changes warrant further investigation to optimize the formulation and ensure safety. Future studies should focus on preclinical AD models to assess the efficacy of nano-lipoxin A4 in reducing cognitive deficits and mitigating neurodegeneration.

## 6. Conclusions

This study successfully developed and characterized Lipoxin A4-loaded nanomicelles, demonstrating their efficacy in modulating amyloid-beta fibrillation in vitro. Nano-lipoxin A4 exhibited a significant dissociative effect on Aβ aggregates, as validated through Thioflavin-T kinetics and electron microscopy, suggesting its potential role in mitigating Aβ pathology in Alzheimer’s disease.

In vivo biodistribution and pharmacokinetic analyses revealed rapid systemic distribution and prolonged elimination of nano-lipoxin A4, with preferential accumulation in the spleen and liver. This behavior aligns with the expected pharmacological profile of lipid-based nanocarriers. However, elevated biochemical markers, particularly liver enzymes, highlight the need for further investigation into the organ-specific metabolism and long-term safety of nano-lipoxin A4.

In conclusion, nanostructured Lipoxin A4 shows promise as a therapeutic strategy for Alzheimer’s disease, particularly in addressing neuroinflammation and amyloid-beta aggregation. Future studies should focus on optimizing the formulation to minimize systemic effects while enhancing therapeutic efficacy.

## Figures and Tables

**Table 1:** Percentage of labeled nano-lipoxin A4 over time, after ascending chromatograms of 99mTc compared to free pertechnetate (Na99mTcO4).

Time (H)	Radiolabeling Efficacy
0	90.73 ± 1.09
1	94.93 ± 0.35
2	99.73 ± 0.07
24	92.32 ± 0.35

**Table 2:** Results of the pharmacokinetic parameters for nano-lipoxin A4 in healthy mice.

Pharmacokinetic Parameters	Nano-Lipoxin (24 h) ± SEM
Concentration at zero time (mg/mL)	4.700 × 10^−11^ ± 2.264 × 10^−12^
Elimination rate/elimination constant (k)	0.0119 ± 0.002668
Volume of distribution (mL)	128.2 ± 6.134
Volume of distribution (L)	0.1282 ± 0.006127
Elimination half-life (1/2) h	63.95 ± 13.52
Elimination half-life (1/2) D	2.664 ± 0.5634
Clearance (L/h)	0.001509 ± 0.0002820

**Table 3:** Results of biochemical analysis of blood plasma. Alanine aminotransferase (ALT), aspartate aminotransferase (AST), gamma GT (GGT), lactate dehydrogenase pyruvate (LDH-P), cholesterol (CHOL), glucose (GLU), creatinine (CRE), lipase (LPS), and amylase (AMS).

Parameters (Units)	Average ± SEM	References ± SEM
ALT (U/L)	83.23 ± 47.20	58.93 ± 9.93
AST (U/L)	32.63 ± 14.19	0.37 ± 0.15
GGT (U/L)	12.47 ± 8.27	5.75 ± 3.95
LDH-P (mg/dL)	1915 ± 552.8	823.8 ± 502.6
CHOL (mg/dL)	275.5 ± 84.36	95.1 ± 11.56
GLU (mg/dL)	311.7 ± 137.2	111.2 ± 9.89
CRE (mg/dL)	0 ± 0	0
LPS (mg/dL)	4667 ± 711.7	1017 ± 542.2
AMS (mg/dL)	571.4 ± 202.2	-

## Data Availability

All data will be available under request.
